# Enhancing Access to Orthopedic Education: Exploring the Potential of Generative Artificial Intelligence (AI) in Improving Health Literacy on Rotator Cuff Injuries

**DOI:** 10.7759/cureus.72833

**Published:** 2024-11-01

**Authors:** Michael J Miskiewicz, Matthew Perez, Salvatore Capotosto, Kenny Ling, Frederick Hance, David Komatsu, Edward D Wang

**Affiliations:** 1 Department of Orthopedic Surgery, Stony Brook University, Stony Brook, USA

**Keywords:** chatgpt, generative artificial intelligence, health literacy, online resources, orthopedic surgery, patient education materials, readability, rotator cuff injury

## Abstract

Introduction: Health literacy plays a vital role in determining one's health status, as studies have shown that poor health literacy is associated with negative health results. The Centers for Disease Control and Prevention (CDC) and the National Institutes of Health (NIH) advise that patient educational materials (PEMs) should be written at an eighth-grade reading level or lower, matching the average reading level of adult Americans. This study evaluated the ability of generative artificial intelligence (AI) to rewrite PEMs about rotator cuff injuries (RCIs) to align with the eighth-grade reading level recommendation of the CDC and NIH.

Methods: Online PEMs about RCI from the 25 highest ranked orthopedic hospitals from the 2022 U.S. News and World Report Best Hospitals Specialty Ranking were collected. Chat Generative Pretrained Transformer Plus, version 4.0 (OpenAI, San Francisco, CA) was then instructed to rewrite the PEMs to adhere to CDC and NIH-recommended guidelines. Readability scores were calculated for the original and rewritten PEMs, and paired t-tests were used to determine statistical significance.

Results: Analysis of the original and rewritten PEMs about RCI demonstrated significant reductions in reading-grade level and word count of 4.33 ± 1.50 (p < 0.001) and 442.68 ± 351.45 (p < 0.001), respectively.

Discussion: Our study determined that generative AI is capable of rewriting PEMs about RCI at a reading comprehension level that conforms to the CDC and NIH guidelines. Hospital administrators and orthopedic surgeons should consider the findings of this study, and the potential utility of AI when crafting PEMs of their own.

## Introduction

Patients can now use the Internet for health-related information, advice, telemedicine consultation, and treatment. The vast repository of online patient educational materials (PEMs) demands continuous monitoring, as the negative implications of misinformation or misinterpretation are profound.

Reports by federal agencies have indicated the average reading grade level for adult Americans hovers around eighth grade [1]. This poses a significant challenge to healthcare providers attempting to explain and educate patients on intricate medical concepts. This knowledge gap has prompted the Centers for Disease Control and Prevention (CDC) and the National Institutes of Health (NIH) to propose guidelines for healthcare providers to use while writing PEMs to mitigate the risk of misinterpretation [2,3]. In short, these guidelines recommend that online PEMs be written at no greater than an eighth-grade reading level, ensuring that a majority of the intended audience will be able to fully comprehend the ideas presented within.

Past research regarding these guidelines has yielded concerning results, with numerous studies documenting a vast discrepancy between the true readability of online PEMs and that recommended by the CDC and NIH [4-6]. These findings are nearly ubiquitous throughout the medical community, and the field of orthopedic surgery is no exception [7-9].

Rotator cuff injuries (RCIs) are some of the most common injuries in orthopedics and sports medicine and are a significant cause of shoulder pain, instability, and reduced range of motion [10,11]. In the general population, an estimated 30% of adults over the age of 60 have a rotator cuff tear [12,13]. As age is one of the most significant risk factors for RCI, its prevalence increases to over 60% of adults over the age of 80 [12,13]. With these data in mind, the need for internet resources to effectively communicate information about the diagnosis and management of RCI cannot be overstated.

Artificial intelligence (AI) has been under development for decades; however, generative AI systems that can create text and images have recently emerged as a powerful new class of AI. Notably, the OpenAI large language model (LLM) Chat Generative Pretrained Transformer (ChatGPT) was first launched in November 2022 [14]. Since then, this deep-learning chatbot (IBM Watson, Yorktown Heights, NY) has continuously expanded in popularity [15]. Recent studies have proposed ways that ChatGPT may be introduced into medical education and practice [16-18], with an underlying emphasis on the LLM's ability to quickly generate responses to questions in a conversational format. Although the system is far from perfect, the continuously improving nature of its generative language fosters significant interest in discovering its full potential in the healthcare space.

The purpose of this study is to explore the potential utility of AI in revising online PEMs related to RCI. We hypothesize that by incorporating several key guideline recommendations made by the CDC and NIH, the AI LLM ChatGPT will be able to rewrite online PEMs from the top 25 nationally ranked orthopedic institutions to an eighth-grade reading level.

## Materials and methods

Patient education material data

The top 25 ranking orthopedic institutions, according to the 2022 U.S. News and World Report Best Hospitals Specialty Ranking, were identified. Each institution's website was queried for PEM related to RCIs. The text within each website was copied and stored. Audiovisual multimedia, including pictures, diagrams, and videos, were not included in the analysis. Exclusion criteria included any institution that did not possess relevant PEM on RCIs.

ChatGPT analysis

The original PEMs were uploaded to ChatGPT Plus, version 4.0 (OpenAI, San Francisco, CA) [14]. ChatGPT was asked to rewrite the PEMs while adhering to the following criteria: 1) limit the total number of polysyllabic words to less than 30, 2) limit sentences to less than 10 words, 3) limit paragraphs to less than five sentences, 4) eliminate as much medical jargon without compromising accuracy, 5) when eliminating medical jargon is not possible, provide a brief explanation of the relevant concept, and 6) overall, rewrite this as if you were speaking to an eighth grader. These parameters were based on pertinent recommendations listed in the CDC's Simply Put and NIH Clear & Simple documents [2,3,7]. A postgraduate year 5 orthopedic surgery resident reviewed all 25 PEMs produced by ChatGPT to ensure the accuracy of the information provided.

Statistical analysis

Readability scores for the original and ChatGPT-rewritten PEMs were calculated using seven unique readability formulas on the ReadabilityFormulas website (MicroPower & Light Co, Dallas, TX) [19]. These formulas include Gunning Fog, Flesch-Kincaid Grade Level, Coleman-Liau Index, Simple Measure of Gobbledygook Index, Automated Readability Index, Linsear Write Formula, and Patrick FORd, John CAylor and Thomas STicht Readability Formula [7]. Each formula analyzes various aspects of a text's composition, such as the number of total words, the number of polysyllabic words, and the complexity of the language used to calculate an overall readability score. These scores correspond to the average reading grade level (Table 1). The average readability score for each institution and the total word count for each PEM were calculated. Paired t-tests were utilized to identify differences between the original PEMs and ChatGPT-rewritten PEMs. SPSS software, version 29.0.0 (241) (IBM Corp., Armonk, NY), was used for all statistical analyses, with p values of 0.05 or less considered statistically significant.

**Table 1:** Description of readability tools and their corresponding formulas SMOG: Simple Measure of Gobbledygook; FORCAST: Patrick FORd, John CAylor and Thomas STicht Source: [7]

Readability tool	Formula
Gunning fog	Grade level = 0.4(average sentence length / percentage of hard words)
Flesch-Kincaid grade level	Grade level = (0.39 × average sentence length) + (11.8 × average # syllable per word) - 15.59
The Coleman-Liau index	Grade level = 0.0855(average # of letters per 100 words) - 0.296(average # of sentences per 100 words) - 15.8
SMOG index	Grade level = 3 × square root of polysyllable count
Automated readability index	Grade level = 4.71 (characters/words) + 0.5 (words/sentences) - 21.43
Linsear Write formula	n = [(2 syllable words × 1) + (3 or more syllable words × 3)] / number of sentences; if n < 20, grade level = n/20 and if n > 20, grade level = n-2/20
FORCAST readability formula	Grade level = 20 - (# of single syllable words × 150 / # of words × 10)

## Results

**Figure 1:**
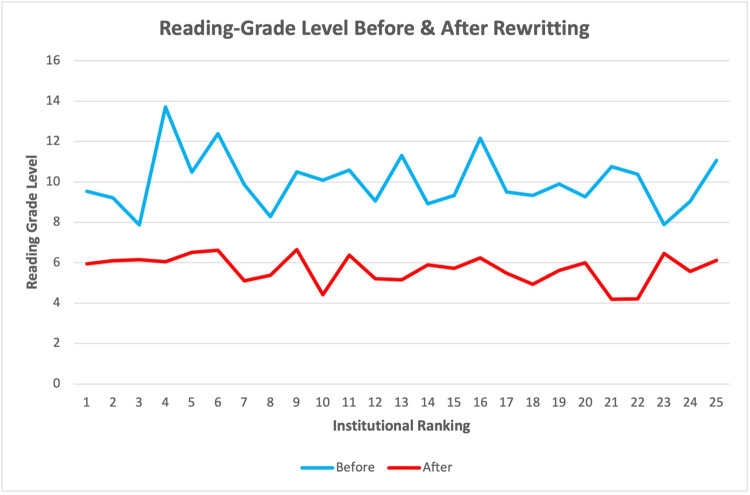
Average readability scores of patient education materials related to rotator cuff injuries from 25 of the top nationally ranked orthopedic institutions, before and after LLM-assisted editing LLM: large language model

PEMs related to RCIs were identified for all 25 orthopedic institutions included in this study. Only two institution's original PEMs demonstrated average readability scores below the recommended eighth-grade reading level. The average readability score across all original PEMs was 10.02 ± 1.86, indicating that most of the content was written at a reading level higher than what is generally recommended for patient education (Figure 1). The average word count of these original PEMs was 755.0 ± 379.76 words.

**Figure 2:**
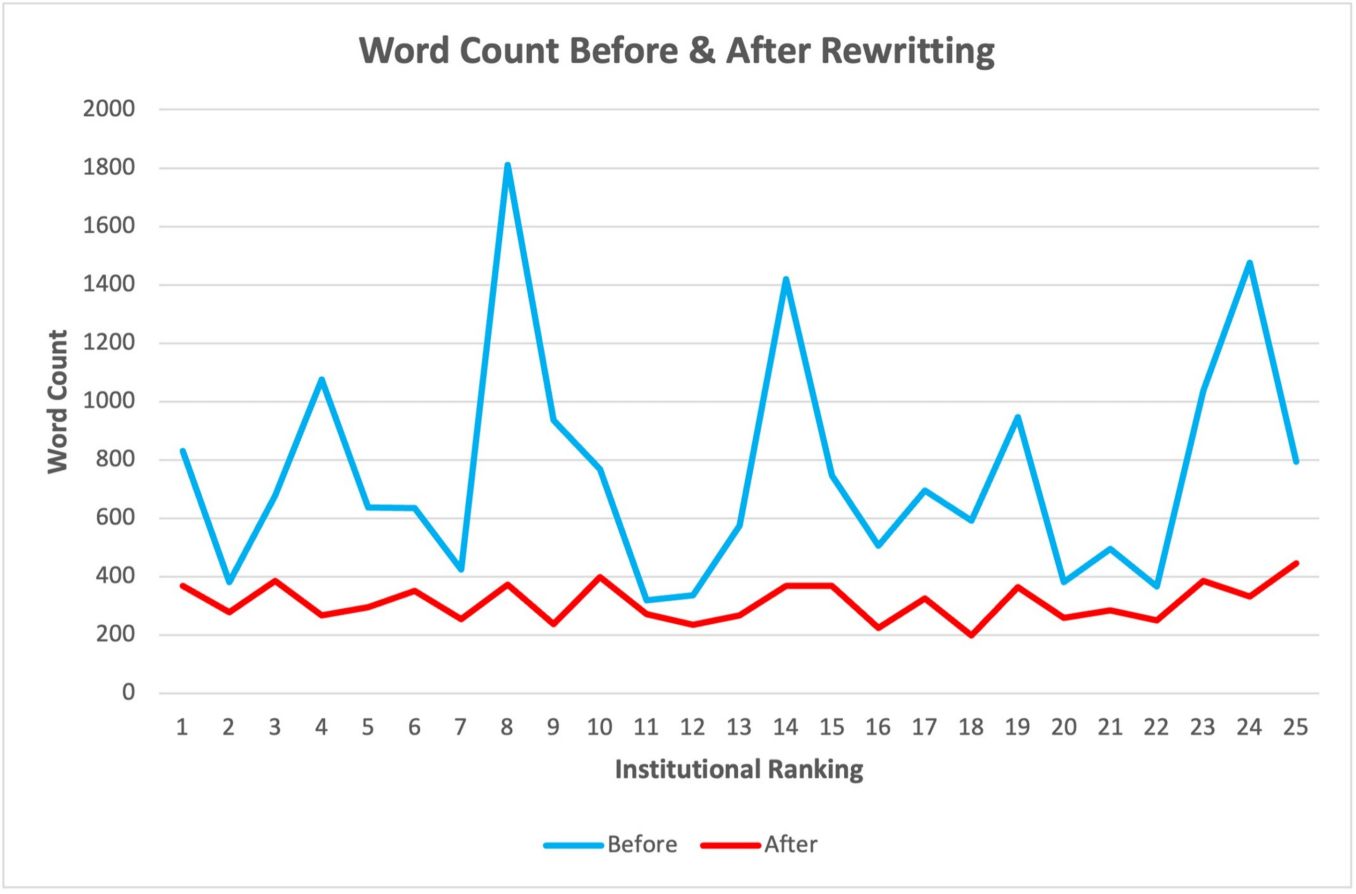
Average word count of patient education materials related to rotator cuff injuries from 25 of the top nationally ranked orthopedic institutions before and after LLM-assisted editing LLM: large language model

Following the application of ChatGPT's rewriting tool, all 25 PEMs were revised to meet readability standards below the eighth-grade level. The average readability score for the rewritten PEMs was 5.67 ± 1.77, reflecting a significant reduction in complexity (Figure 2). Furthermore, the revised PEMs exhibited a more concise format, with an average word count of 312.2 ± 65.73, showing a marked decrease in length compared to the original versions.

Figure 1 provides a visual comparison of the readability scores before and after the revisions, demonstrating a clear improvement in accessibility. The original PEMs had a wider range of readability scores, while the rewritten materials consistently achieved scores at or below the eighth-grade reading level. Figure 2 illustrates the reduction in word count across all institutions' PEMs, with a sharp decline in average word count after the revisions.

Table 2 summarizes the key statistical findings from the study, showing a significant decrease in both the average readability score and the average word count of the PEMs. The average reduction in reading grade level was 4.33 ± 1.50 (p < 0.001), indicating a substantial improvement in the readability of the materials. In terms of word count, there was an average reduction of 442.68 ± 351.45 words per PEM (p < 0.001), making the rewritten versions more concise and likely easier for patients to digest.

**Table 2:** Results for paired t-test to determine the significance between average readability scores and average total word count of the original and rewritten PEMs PEMs: patient educational materials

Metric	Mean reduction	Standard deviation	95% confidence interval		p value
			Lower	Upper	
Readability score	4.33	1.50	3.72	4.95	<0.001
Word count	442.68	351.46	297.60	587.76	<0.001

The accuracy of the medical information within the ChatGPT-rewritten PEMs was verified by a senior orthopedic resident to ensure that the content remained clinically relevant and reliable after simplification. An example of an original and ChatGPT-rewritten PEM is provided in the Appendix.

## Discussion

Our study demonstrates that PEMs related to RCIs, available on the websites of the top 25 nationally ranked orthopedic institutions, are written at a level of complexity that far surpasses the recommended level set forth by the CDC and NIH. This initial finding is not surprising or unique, as there is a significant body of research demonstrating similar findings in both orthopedic and nonorthopedic domains [4-9]. In light of this, we explored a potential solution to this widespread problem. Through the guidance of recommendations set forth by the CDC and NIH, we demonstrate that an LLM is capable of rewriting online PEMs so that they may be easily understood by the majority of American adults. Specifically, we found that ChatGPT Plus (version 4.0) was able to revise PEMs related to RCI, published online by the top 25 ranked orthopedic institutions, and reduce the average readability scores and word count of RCI PEMs by 43.1% and 58.7%, respectively. The outcomes of our study stress the potential utility of AI in making online health information more accessible and understandable.

Recent studies have suggested that ChatGPT holds considerable potential in the healthcare field [16-18,20]. ChatGPT has achieved passing scores (60%) on the United States Medical Licensing Examinations and several other specialty-specific board examinations, suggesting the AI model's potential utility as an educational tool [20-22]. Furthermore, a recent study by Rao et al. attempted to assess the LLM's capabilities as it relates to radiologic decision-making [23]. Considerable advancement in AI technology must be made before ChatGPT and other LLMs can be consistently relied upon for clinical decision-making; however, studies such as these underscore a growing ability to integrate AI into a physician’s daily workflow.

To date, limited studies have explored LLMs' potential use to aid writing PEMs in the orthopedic realm. One study by Fahy et al. investigated ChatGPT's ability to answer common patient queries related to anterior cruciate ligament injury and surgery [9]. The LLM was found capable of providing detailed responses to patient queries. However, the accuracy of the information provided and the information's complex readability were notable limitations. This study highlights two important pitfalls of LLMs. First, the developers of ChatGPT have cautioned users that the LLM notoriously produces seemingly plausible but factually incorrect information [14,15]. Second, without proper guidance, ChatGPT will not prioritize the readability of its output. The current study attempts to address these shortcomings of the LLM. By instructing ChatGPT to rewrite previously verified PEMs, we mitigate the risk of ChatGPT incorporating novel erroneous information. Furthermore, by providing ChatGPT with clear guidelines on how to improve the PEM's readability, we ensured the material was written at a level of complexity that would be easily understood by the vast majority of Americans. Our approach proposes a detailed and effective strategy for improving the readability of PEMs. Such methods may demonstrate a fruitful improvement in the accessibility and dissemination of health information if applied to current online educational content.

Limitations

We acknowledge certain limitations of this study. First, the seven readability formulas implemented during this study are widely utilized to estimate the educational level required to understand a text. However, they primarily evaluate readability based on quantifiable elements like sentence length, characters per word, and word complexity. The reliance on structural aspects of a language's text may fail to acknowledge the influence of other key factors that determine readability, such as the organization of information presented, the language the information is written in, and the use of supplemental pictures, diagrams, and videos. Furthermore, ChatGPT is not able to analyze pictures, videos, and other forms of multimedia. This limitation of ChatGPT's capabilities hinders its ability to comprehensively revise all aspects of the PEMs' readability.

Despite these limitations, this study successfully addressed the decades-long problem of poor readability of PEMs within the healthcare sector. Potential avenues for future research include exploring alternative LLMs that can better evaluate the impact of audiovisual multimedia on the readability of PEMs, as well as comparing the readability of PEMs written in different languages to uncover any cultural or linguistic barriers within patient education resources.

## Conclusions

This study explored ChatGPT's ability to revise online PEMs on RCI to meet the readability levels recommended by the CDC and NIH. Our findings emphasize the potential role ChatGPT can have in enhancing the dissemination of health information through online PEMs. Orthopedic surgeons may consider the findings of this study when crafting PEMs of their own to improve patient education and outcomes.

## Appendices

**Table 3:** Original and ChatGPT rewritten online patient education material example ChatGPT: Chat Generative Pretrained Transformer Source: [24]

Original	ChatGPT Edited
What is a rotator cuff? The rotator cuff of the shoulder is made up of four muscles. Tendons of these muscles come together to form a covering around the head of the upper arm bone (humerus) and top of the shoulder. The rotator cuff muscles are important stabilizers and movers of the shoulder joint. As the name implies, the rotator cuff functions to allow you to rotate your shoulder and lift your arm. What are common rotator cuff injuries? Common rotator cuff injuries include rotator cuff tendonitis and rotator cuff strain, which is a partial or complete tear of the rotator cuff. Rotator cuff tendonitis is inflammation or irritation in the tendons and cuff muscles that help move your shoulder joint. This injury typically occurs over time, usually as a result of keeping your shoulder in a single position for a prolonged period (such as sleeping on your shoulder every night), by overhead work-related activities, or athletic activities such as tennis, baseball, cricket or jai alai. A rotator cuff tear may be partial or complete. A partial tear is when one of the tendons of the rotator cuff is frayed or damaged. A complete tear (also called a full-thickness tear) is when the tendon in is severed in half or pulled completely off of the bone. Rotator cuff tears often occur over time from prolonged wear and tear. However, the rotator cuff may also be acutely injured by trauma involving a fall on the arm and shoulder or from heavy lifting. (It is common among weightlifters.) A dislocated shoulder injury may also cause a torn rotator cuff. What are the symptoms of rotator cuff tendonitis? Rotator cuff tendonitis is the inflammation or irritation of the tendons and muscles in the shoulder joint. Symptoms of rotator cuff tendonitis typically get worse over time. These symptoms may include: pain or swelling in the front of the shoulder pain or swelling in the side of the arm pain when raising or lowering the arm clicking or popping sound when the arm is moved pain that disrupts sleep loss of mobility or strength in affected arm What are the symptoms of a torn rotator cuff? Symptoms of a partial or complete torn rotator cuff may include: pain in the front and/or down the outside of the shoulder having trouble raising the arm experiencing weakness in the shoulder feeling pain when the arm is moved in certain ways being unable to lift things hearing clicking or popping when the arm is moved How are rotator cuff injuries diagnosed? A doctor will first take the patient’s medical history and perform a physical examination to: locate areas of shoulder pain or tenderness test the range of motion and, possibly, the strength of the shoulder joint If a tear is suspected, an X-ray and/or MRI may be ordered. When diagnosing a shoulder cuff injury, a doctor will also rule out conditions that can cause similar symptoms, such as a compressed nerve in the neck (called a "pinched nerve") or shoulder arthritis. How is rotator cuff tendonitis treated? The treatment for rotator cuff tendonitis includes first reducing swelling and inflammation then strengthening muscles and improving range of motion. Treatments may include: rest NSAIDs (nonsteroidal anti-inflammatory drugs) corticosteroid injections platelet-rich plasma (PRP) injections All of the above treatments are usually followed by supervised physical therapy to regain shoulder motion and strength. How is a rotator cuff tear treated? Mild rotator cuff tears may be treated nonsurgically with anti-inflammatory medication, steroid injections and/or physical therapy. However, for more severe tears or for active individuals who engage in sports or overhead work, surgery is often recommended. A partial or complete tear of the rotator cuff tendon is generally repaired by arthroscopic surgery. Open surgery (using a larger incision) may be necessary for large, complicated full tears of the rotator cuff. When, years after a rotator cuff tear, a person develops severe shoulder arthritis, they may benefit from a surgery called reverse shoulder replacement. How long does it take to recover from a rotator cuff injury? The minimum recovery time for rotator cuff tendonitis is generally two to four weeks. Left untreated, rotator cuff tendonitis can worsen and may lead to a partial or complete rotator cuff tear. Symptoms of a partial or complete rotator cuff tear can last from several weeks up to several months depending on the severity of the injury and the treatment plan. When should I see a doctor for a rotator cuff tear? You should see an orthopedic surgeon or sports medicine doctor if pain from a rotator cuff injury is affecting your daily life, waking you up from sleep or reducing your range of motion. Make an appointment to see a doctor if: Your shoulder hurts when you do the same activity over and over. You experience a loss in range of motion. Shoulder pain awakens you from sleep. Shoulder pain is accompanied by swelling, redness, or tenderness around the joint.	Rotator Cuff 101 **What Is a Rotator Cuff?** Your rotator cuff is in your shoulder. It's made of four muscles and their tendons. They wrap around the top of your arm bone and shoulder. It helps you move and rotate your arm. **Common Injuries** You can get rotator cuff tendonitis or a tear. Tendonitis means the tendons are irritated. This can happen from doing the same thing a lot, like playing tennis or painting overhead. A tear means the tendon is partly or completely ripped. This can happen slowly from using it a lot or suddenly from a fall or lifting something heavy. **Symptoms to Watch For** With tendonitis, your shoulder might hurt or swell. It can hurt more when you move your arm, or even wake you up. A tear can make your shoulder hurt, feel weak, or make a popping sound. It might be hard to lift things or move your arm in certain ways. **How to Find Out What's Wrong** A doctor will ask about your pain and check how you move your arm. You might need an X-ray or MRI to see the injury better. **Fixing Tendonitis** To treat tendonitis, you might need to rest, take medicine to lower swelling, get shots to ease pain, or try special injections to heal the tendons. Physical therapy can also help make your shoulder stronger and move better. **Fixing a Tear** For a small tear, you might just need medicine, shots, or therapy. If it's a big tear or you're very active, you might need surgery. Surgery can fix the tear with a few small cuts or a big one, depending on the tear size. If your shoulder gets really arthritic from an old tear, there's a surgery that can replace the shoulder in a new way. **Recovery Time** Tendonitis might take a few weeks to get better. A tear could take weeks to months, depending on how bad it is and if you have surgery. **When to See a Doctor** If your shoulder hurts a lot, wakes you up, or you can't move it like before, see a doctor. Especially if it hurts doing the same things over and over, feels stiff, or looks swollen.
